# Predictors of enophthalmos among adult patients with pure orbital blowout fractures

**DOI:** 10.1371/journal.pone.0204946

**Published:** 2018-10-05

**Authors:** Suraya Ahmad Nasir, Roszalina Ramli, Nazimi Abd Jabar

**Affiliations:** Centre for Oral & Maxillofacial Surgery, Faculty of Dentistry, Universiti Kebangsaan Malaysia, Jalan Raja Muda Abdul Aziz, Kuala Lumpur, Malaysia; Navodaya Dental College and Hospital, INDIA

## Abstract

The aim of this study was to determine the predictors of post-traumatic enophthalmos (PE) in relation to the internal orbital changes following pure orbital blowout fractures. The design was a 10-year retrospective cross-sectional study analysing 629 medical records and computed tomography (CT) data of patients with orbital fractures from January 2008 to January 2017. Demographic, etiology, co-morbidity and clinical characteristics were obtained from the medical records. Assessment of the PE, fracture site and size, intraorbital structures and muscle change were performed using the Digital Imaging and Communications in Medicine (DICOM) viewer software, OsiriX v5.8.2. Of the 629 patients with orbital fractures, 87 were pure orbital blowout fractures. Demographic pattern showed that males outnumbered females in the series, with male: female ratio of 5.7:1. The mean age was 37.2 ± 14.7 and the main etiology was motor vehicle accident. Orbital floor fracture was the most common fracture location (67.8%). The involvement of the posterior ledge and inferior orbital fissure showed statistical significant difference with PE (Fisher’s exact test, p = 0.03). Binary logistic regression showed that after controlling for age, patients with fracture size of more than 150 mm^2^ had three times the odds of sustaining a PE, (adjusted odds ratio (AOR) = 3.01 (95% CI 1.17–7.92).

Fracture size larger than 150 mm^2^ was a radiological predictor of PE. Additional research investigating further on the role of concurrent fracture of the posterior ledge and inferior orbital fissure is advocated.

## Introduction

Pure orbital blowout fracture confines within the internal orbital wall [1–3]. It does not involve the orbital rim or other facial bones. Posttraumatic enophthalmos (PE) was described as the most debilitating complication of this fracture [4]. PE is clinically characterized as backward displacement of the eyeball. This could lead to motility disturbances and diplopia. High incidence of PE that ranged from 30% to 62% had been reported [5–8]. Generally, orbital floor fracture of less than 50% in size rarely causes a PE, but a combined fracture of the floor-medial wall results in prominent PE [9–11].

PE is assessed clinically using the Hertel exophthalmometer. 2 mm or more in difference in the axial displacement between the two globes is considered to be clinically significant [12,13] and this would indicate for a surgical intervention. The other assessment is through the computed tomography (CT) images as described by Lee and Lee [14].

Many studies have been conducted and showed intriguing evidence in predicting the risks of a PE. Factors such as location of the fracture [3,15], fracture size [16–18] and muscle change [19,20] have been identified as the predictors of PE. However, there is a paucity in the literature in relation to all these factors being analysed simultaneously. We therefore conducted this study with the objectives as below:

- to determine the prevalence of pure orbital blowout fracture observed in selected tertiary hospitals within one geographical area in Malaysia
- to explore the association between the fracture site, size, medial and inferior rectus muscle change, and the involvement of intraorbital structures with PE.

## Materials and methods

### Ethical approval

Approval from the ethics committee of each center was obtained i.e.:

- Research Ethics Committee, The National University of Malaysia [UKM 1.5.3.5/244/DD/2015/011(2)];
- Medical Research and Ethics Committee, National Institutes of Health, Ministry of Health Malaysia [NMRR-16-B40-29119(IIR)];
- Medical Ethics Committee, Faculty of Dentistry, University of Malaya [DF 0S1601/0001(P)];
- Medical Ethics Committee University Malaya Medical Center [MECID. NO: 20162–2195].

The research was conducted in accordance to the Declaration of Helsinki. The requirement for informed consent was waived by the above ethic committees.

### Data collection

Medical records of patients from three selected hospitals in Klang Valley, Malaysia, who sustained orbital fractures between 1^st^ January 2008 to 31^st^ January 2017 were reviewed. The inclusion criteria were adult patients aged 18 years old and above. This age group was selected based on the growth of the orbit to adult size as well as the adult pattern of fracture. The growth to adult size was reported to be between 11 to 15 years old [21,22]. In relation to the pattern of fracture, a trap-door or pediatric/adolescent type was shown to occur below the age of 18 years [23]. Hence 18 years was selected as the minimum age limit.

The patients must have a post-trauma CT scan of at least 2 mm slice interval.

Patients with impure orbital fractures, pre-existing enophthalmos or non-intact globe were excluded from the study.

#### Variables of interest

The outcome or dependent variable was the post-traumatic enophthalmos (PE) while the independent variables included demographic and injury characteristics.

The assessment of the variables is described as below.

#### i) Independent variables

Digital Imaging and Communications in Medicine (DICOM) data were analyzed using the OsiriX v5.8.2 software (Pixmeo, Geneva, Switzerland). The CT analyses included measurement of the (a) fracture size (b) involvement of the intraorbital structures (c) fracture site and (d) extrinsic muscle shape change (height to width ratio)[19] and this was compared with the normal contralateral orbit. The classification of the fracture site was modified from the comprehensive *Arbeitsgemeinschaft für Osteosynthesefragen Craniomaxillofacial* (AOCMF) classification system by Kunz et al. [24].

#### a) Measurement of the fracture size

**Fig 1:**
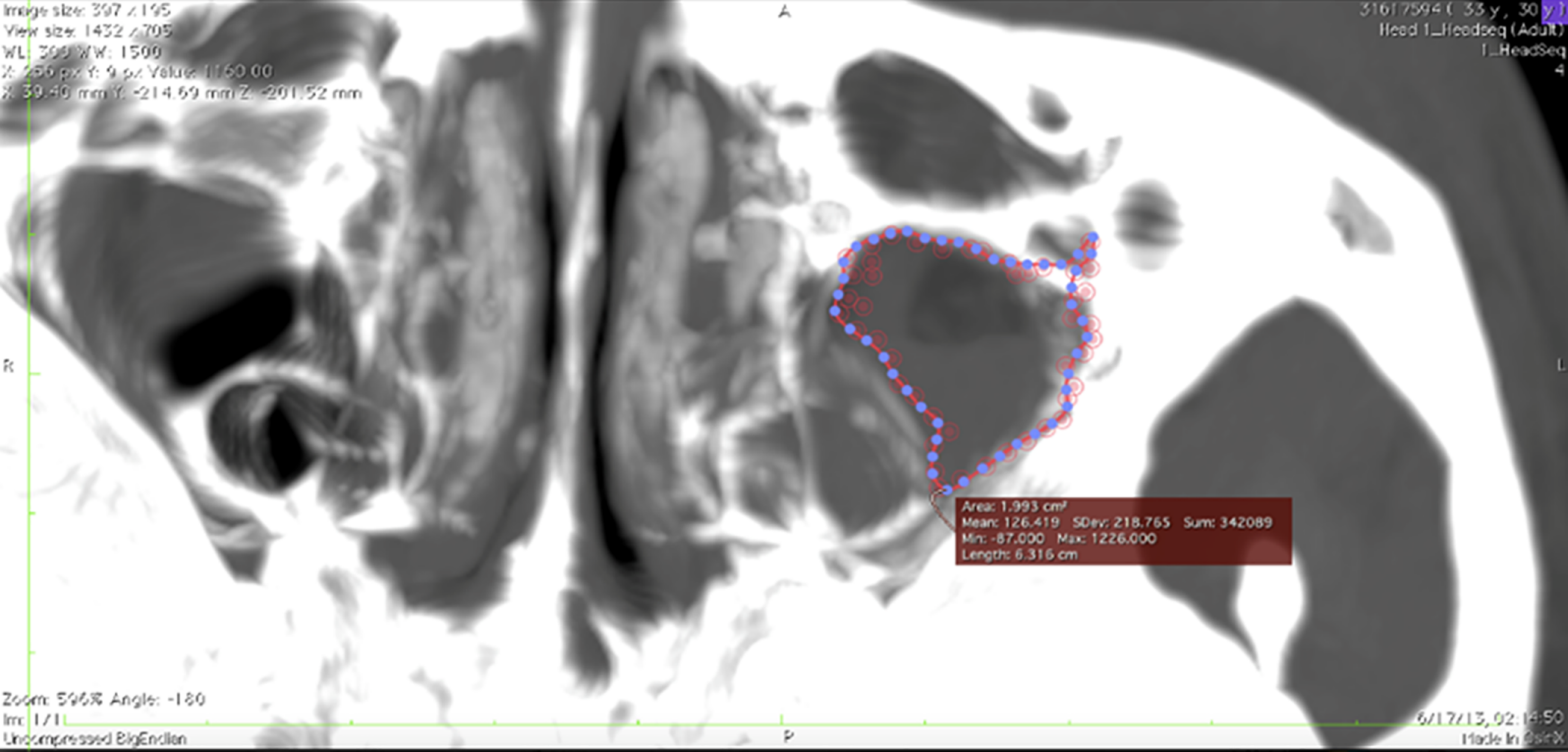
The axial computed tomography with area of fracture. The area of fracture is automatically calculated using the pencil tool in the OsiriX v5.8.2 software.

Measurement of the fracture size was performed based from Ang et al. (2015) [25]. The measurement started at the coronal view of the scan. The area of interest was identified from anterior to the posterior region. The point tool was used to mark the area of involvement. The axial images were then reconstructed from the coronal images using the 3-Dimensional (3D) Multiplanar Reconstruction (MPR) mode (Fig 1). All the images were stacked onto each other by increasing the slab thickness to more than 20 mm. A single image with the most points was used for measurement.

#### b) Identification of the intraorbital structures

**Fig 2:**
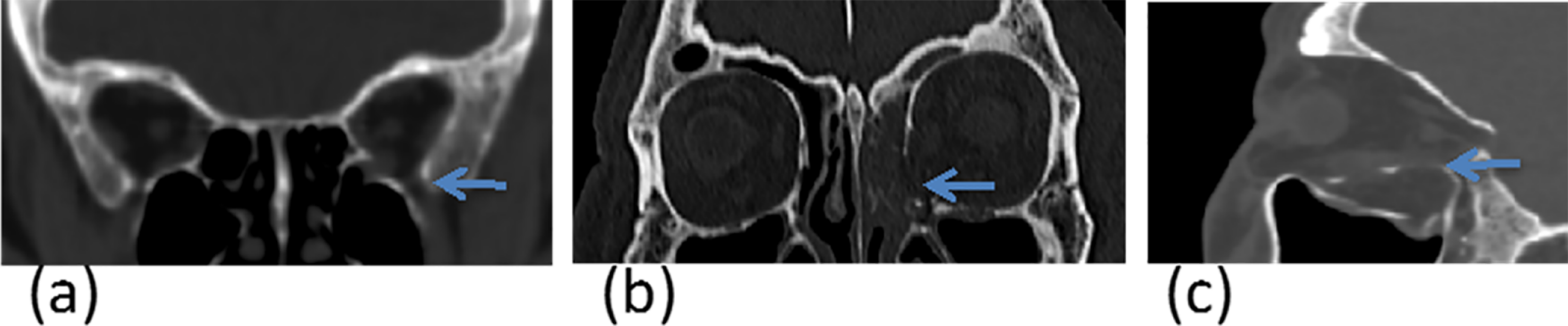
**The intraorbital structures in coronal (a and b) and sagittal view (c).** The involvement of intraorbital structures, including (a) inferior orbital fissure, (b) intraorbital buttress, and (c) posterior ledge.

Important intraorbital structures were identified based on Kunz et al. (2014) [24]. These included the inferior orbital fissure, intraorbital buttress and the posterior ledge (Fig 2). This study focused on fracture of the orbital floor, medial floor and combination of orbital floor and medial wall with or without the involvement of the intraorbital buttress.

#### c) Measurement of the muscle change

**Fig 3:**
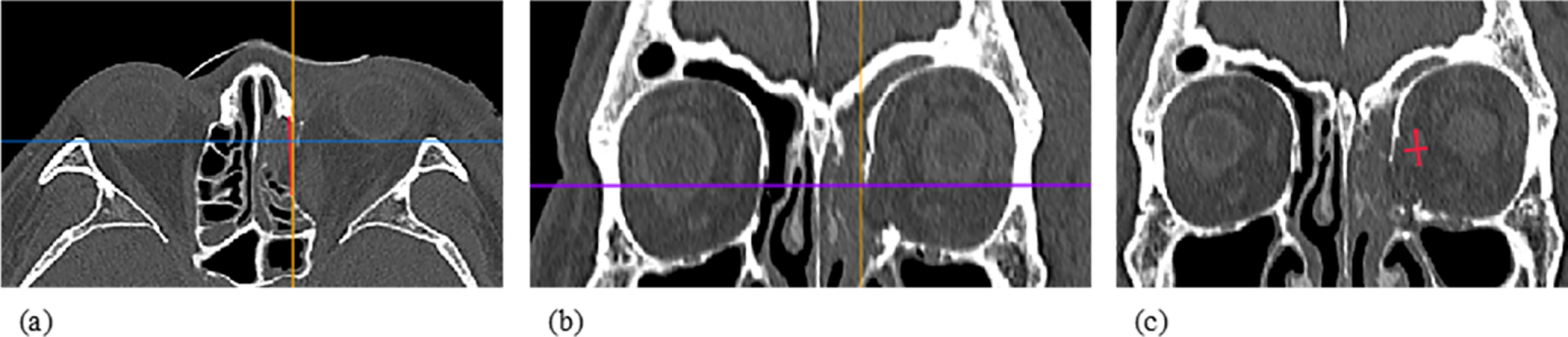
Measurement of medial rectus muscle changes. Identification of the medial wall fracture in (a) axial view at the level of optic canal and (b) in coronal view. (c) Calculation of the muscle ratio.

The muscle ratio calculation was conducted following identification of the fracture site. The sagittal view was used to identify the inferior rectus muscle that was associated with the fracture in the regions of ID 10, 16 and ID 11 (original locations as described by Kunz et al. [24]. As for the medial rectus muscle that was in association with the ID 9 and ID 15 [24], the axial view was used to identify the fracture site. A parallel line was placed from the anterior to the posterior defect margin. The muscle ratio was then calculated in three different measurement areas on the coronal view based on the total anteroposterior fracture defect. The height to width ratio of the inferior and medial rectus muscle was measured in the fractured orbit and compared with the normal orbit (Fig 3). The maximum ratio changes and fracture size variables were used in the data analysis [19].

#### ii) Dependent variable: the PE

Assessment of the PE was made clinically by the respective surgical teams in the hospitals and we used the findings that were documented in the medical records. The PE was validated by the CT scan.

#### Measurement of the PE

**Fig 4:**
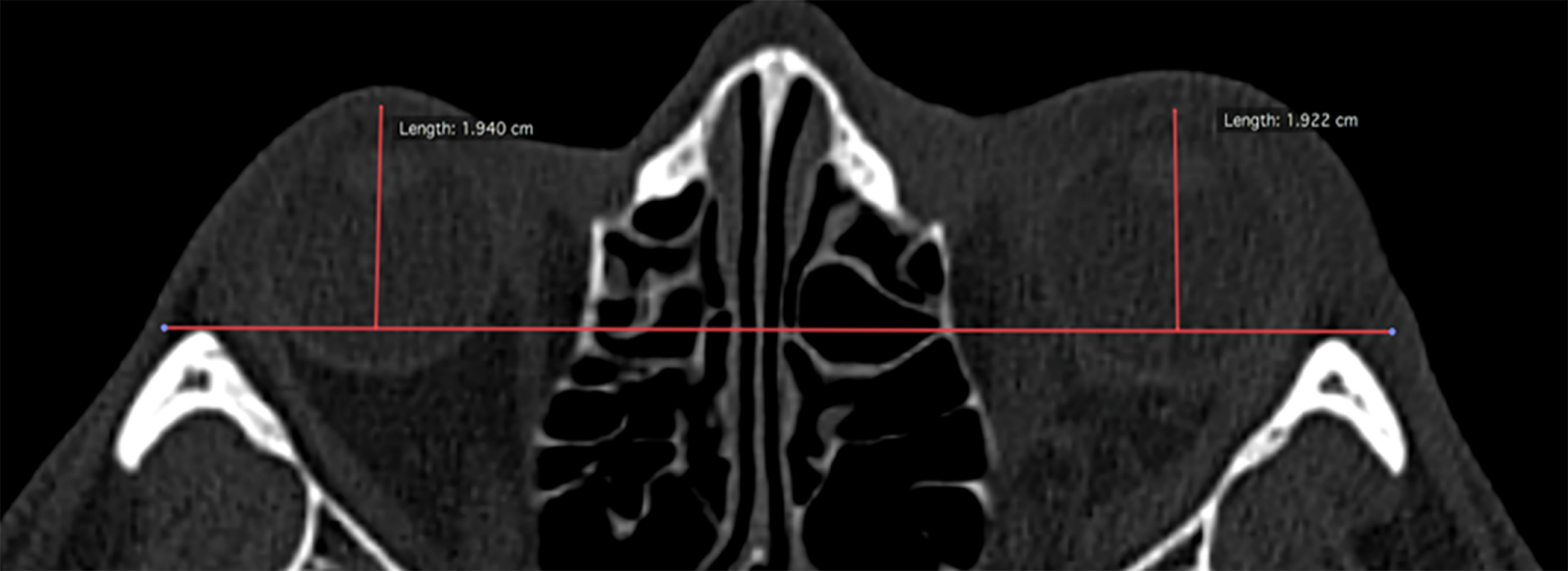
Measurement of enophthalmos. A horizontal line (baseline) is drawn to connect the outermost of the bilateral anterior tips of the lateral walls. A vertical line is then drawn from the most prominent corneal part to the baseline. The length of this line is measured. The difference in the measurement value between the normal and the affected orbit is used to conclude the presence or absence of PE, or proptosis of the orbit.

Measurement of PE was carried out using the axial CT scan view. The skull was aligned according to both Frankfort and axial plane. The anterior orbital boundary was defined as the horizontal line that connected the outermost of bilateral anterior tips of the lateral walls. The posterior boundary was defined as the anterior aspect of the optic canal. A line connecting bilateral prominent points of the lateral edge was made. The centre of the cornea was identified and a vertical line was drawn to connect it perpendicularly with the first line. The measurement of the PE was the difference between both perpendicular lines [26](Fig 4). According to Whitehouse et al. (1994), every cm^3^ increase in volume represented approximately 0.77 mm of PE [8]. Thus, radiographical PE was considered to be present if the measurement was above 0.77 mm [8]. Measurement protocol was planned to synchronize the radiographical measurement. Kappa statistic was calculated for two examiners for calibration purpose. Once good value was achieved, i.e. no difference between observers’ measurement of the PE at both right and left orbits, one researcher proceed to measure the radiographical PE in all the remaining CT scans.

### Statistical analyses

All statistical analyses were performed using IBM SPSS Statistics version 23.0 (Armonk, NY: IBM Corp.). Descriptive analysis such as frequencies, percentages and mean with standard deviation (SD) or median with interquartile (IQR) was used where appropriate. Measurement of the radiological study factors was performed by two independent examiners. Kappa statistic was calculated for two examiners. Good agreement was achieved when the value was 0.60–1.00.

Pearson’s chi-square test and Fisher’s exact test were used to determine the association between the clinical characteristics of the pure orbital blow out fracture and PE. Non-parametric Mann-Whitney U test was used to determine the association between the fracture location and size, and inferior and medial rectus muscle changes with PE. Multivariate analysis was performed to determine the clinical predictors of PE among the pure orbital fracture cases. Selection of potential predictors with p <0.25 [27] were made as well as those with clinical relevance.

Final logistic regression model was achieved by adding the independent variables into the model using the automated backward conditional method. The results was presented in the form of odds ratios (OR) with 95% Confidence Interval (CI).

Quality of the model was assessed using the classification table for accuracy and the Hosmer-Lemeshow test for goodness of fit. The significance level for all the tests was set at *p* <0.05.

## Results

### Prevalence and demographic factors

Pure orbital blowout fracture was diagnosed in 87 patients among 629 individuals who sustained orbital fractures. The prevalence was 13.8%.

**Fig 5:**
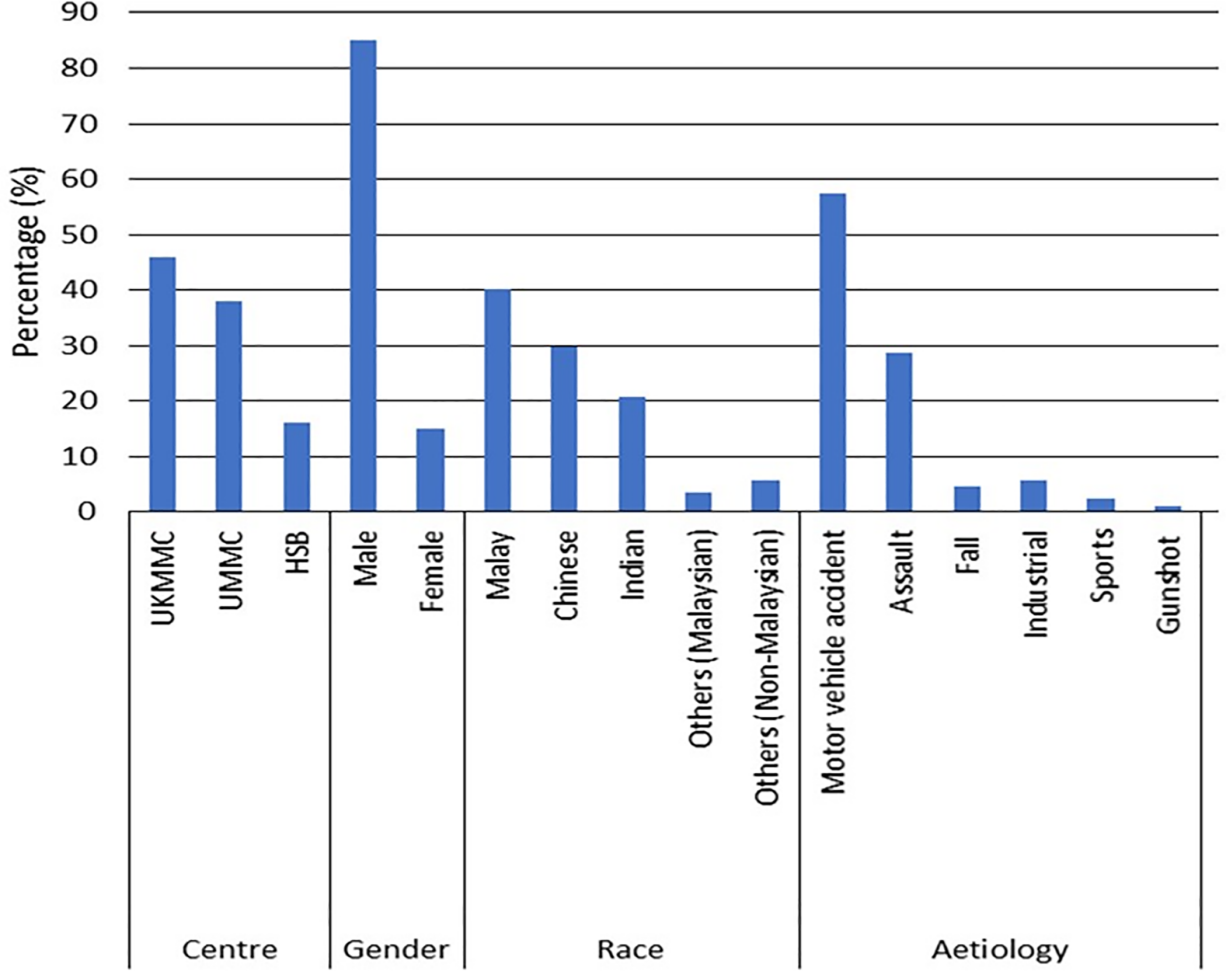
Demographic factors and etiology of pure orbital fractures. Fig 5 showing descriptive statistics (in percentage) for the occurrence of pure orbital blow out fractures in each hospital and according to race, gender and etiology.

The mean age was 37.2 ± SD 14.7 years (range 19–74 years) with male: female ratio of 5.7:1. Majority of the patients were Malays (40%), followed by Chinese (30%) and Indians (20%). More than half of the cases were due to motorvehicle accident (MVA) (57.5%), assaults (28.7%) others (Fig 5).

### Characteristics of pure orbital blowout fractures

Majority of the pure orbital blowout fractures involved the orbital floor (67.8%). Both the posterior ledge (31%) and inferior orbital fissure (24.1%) showed high rate of involvement compared to other internal orbital structures, alone or in combination (Table 1). The largest fracture size was observed when there was a continuous fracture in the floor and the medial wall (mean ± SD: 373.9 ± 109.1 mm).

**Table 1:** Radiographical characteristics of the pure orbital blowout fracture.

Radiographical Characteristics	Total, n (%) (N = 87)
**Internal orbital structure involvement**	
**Intraorbital buttress**	
Fracture	8 (9.2)
No fracture	79 (90.8)
**Posterior ledge**	
Fracture	27 (31.0)
No fracture	60 (69.0)
**Inferior orbital fissure**	
Fracture	21 (24.1)
No fracture	66 (75.9)
**Internal orbital structure involvement (in combination)**	
Intraorbital buttress	3 (3.4)
Posterior ledge	18 (20.7)
Inferior orbital fissure	12 (13.8)
Intraorbital buttress and posterior ledge	1 (1.1)
Intraorbital buttress and inferior orbital fissure	1 (1.1)
Posterior ledge and inferior orbital fissure	5 (5.7)
Intraorbital buttress, posterior ledge and inferior orbital fissure	3 (3.4)
**Fracture site**	
Floor only	59 (67.8)
Medial wall only	9 (10.3)
Floor and medial wall	17 (19.5)
Floor continuous with medial wall	2 (2.3)
**Fracture size (mm**^**2**^**)**	
**Floor only**	
Mean ± Standard deviation	145.2 ± 64.0
Min	45.6
Max	377.3
**Medial wall only**	
Mean ± Standard deviation	45.3 ± 20.6
Min	21.6
Max	79.4
**Floor and medial wall (as a separate fracture)**	
Floor	
Mean ± Standard deviation	121.9 ± 60.4
Min	32.8
Max	244.7
Medial wall	
Mean ± Standard deviation	43.4 ±19.5
Min	20.7
Max	80.0
**Floor and medial wall (as a combined fracture)**	
Mean ± Standard deviation	373.9 ± 109.1
Min	296.7
Max	451.0

### Clinical and radiographic PE measurement

The PE was documented positive clinically in 33.3% of the patients with majority detected between 6 to 20 days post injury (mean 16.0 ± SD 15.7 days).

Using the CT scans, the PE was identified in 37.9% of the patients, and most of the CT scans were conducted on the first day of trauma (mean 2.7 ± SD 5.7 days).

### Univariate analyses

#### Internal orbital structure involvement and PE

A total of 16 patients (18.4%) with minimum fracture of one of the internal orbital structures had PE. From this subset, 6.9% of patients with two or more sites of internal orbital structures’ fractures presented with PE. The involvement of the posterior ledge and inferior orbital fissure showed statistical significant difference with PE (Fisher’s Exact Test, *p* = 0.03) (Table 2).

**Table 2:** Association between the fracture of internal orbital structures, fracture location and muscle change with PE.

Characteristics	Total, n (%) (N = 87)	PE, n (%)		*p*-value
		With (n = 29)	Without (n = 58)	
**Internal Orbital Structures**				
**Intraorbital buttress**				
Intact	79 (90.8)	26 (32.9)	53 (67.1)	1.00
Fractured	8 (9.2)	3 (37.5)	5 (62.5)	
**Posterior ledge**				
Intact	60 (69.0)	18 (30.0)	42 (70.0)	0.33
Fractured	27 (31.0)	11 (40.7)	16 (59.3)	
**Inferior orbital fissure**				
Intact	66 (75.9)	20 (30.3)	46 (69.7)	0.29
Fractured	21 (24.1)	9 (42.9)	12 (57.1)	
**Intraorbital buttress & posterior ledge**				
Not involved	86 (98.9)	29 (33.3)	5 (65.5)	1.00
Involved	1 (1.1)	0 (0)	1 (1.1)	
**Intraorbital buttress & inferior orbital fissure**				
Not involved	86 (98.9)	29 (33.3)	57 (65.5)	1.00
Involved	1 (1.1)	0 (0)	1 (1.1)	
**Posterior ledge & inferior orbital fissure**				
Not involved	82 (94.3)	24 (27.6)	58 (66.7)	0.03
Involved	5 (5.7)	5 (5.7)	0 (0)	
**All structures**				
Not involved	84 (96.6)	28 (32.2)	56 (64.4)	1.00
Involved	3 (3.4)	1 (1.1)	2 (2.3)	
**Fracture Location**				
**Orbital Floor**				
**ID 10**				
Intact	29 (33.3)	8 (9.2)	21 (24.1)	0.42
Fractured	58 (66.7)	21 (24.1)	37 (42.5)	
**ID 16**				
Intact	26 (29.9)	8 (9.2)	18 (20.7)	0.74
Fractured	61 (70.1)	21 (24.1)	40 (46.0)	
**ID 11**				
Intact	38 (43.7)	9 (10.3)	29 (33.3)	0.09
Fractured	49 (56.3)	20 (23.0)	29 (33.3)	
**Medial Orbital Wall**				
**ID 9 & 15**				
Intact	59 (67.8)	19 (21.8)	40 (46.0)	0.75
Fractured	28 (32.2)	10 (11.5)	18 (20.7)	
**Muscle Change**				
**Inferior rectus muscle**				
**ID 10**				
Yes	8 (9.2)	3 (3.4)	5 (5.7)	1.00
No	79 (90.8)	26 (29.9)	53 (60.9)	
**ID 16**				
Yes	21 (24.1)	9 (10.3)	12 (13.8)	0.29
No	66 (75.9)	20 (23.0)	46 (52.9)	
**ID 11**				
Yes	23 (26.4)	10 (11.5)	13 (14.9)	0.23
No	64 (73.6)	19 (21.8)	45 (51.7)	
**Medial rectus muscle**				
**ID 9 & 15**				
Yes	11 (12.6)	5 (5.7)	6 (6.9)	0.50
No	76 (87.4)	24 (27.6)	52 (59.8)	

Note: ID 10 = anterior third of orbital floor; ID 16 = middle third of orbital floor; ID 11 = anterior third of orbital floor (including part of zygoma); ID 9 and 15 = medial orbital wall.

#### Fracture location and PE

All the selected fracture locations showed high percentage of being fractured compared to being intact, except for the combination location ID 9 and 15 (original locations as described by Kunz et al.[24]) on the medial wall of the orbit (Table 2). When analysed individually, Pearson chi square test did not show any significant difference between the selected fracture locations (the ID 10, 16, 11, 9 and 15) and PE (Pearson Chi Square, *p* > 0.05).

#### Fracture size and PE

The size of the fracture locations did not significantly related with a PE (Mann-Whitney U Test, *p* > 0.05) (Table 3). No patient with isolated medial wall fracture showed evidence of a PE. The median latencies were larger in patients without a PE in the floor and medial wall fractures with an intact internal orbital buttress. No comparison could be made on patients with continuous fracture of the orbital floor and medial wall due to low sample size.

**Table 3:** The association between fracture size (mm^2^), fracture locations, muscle change and PE.

Characteristics	PE, median (IQR)		*U—*value	*p—*value
	With	Without		
**Fracture size**				
Floor only (n = 59)	154.7 (71.3)	133.6 (79.0)	280.0	0.11
Medial wall only (n = 9)	NA	38.6 (39.2)	3.0	0.70
Floor and medial wall (as a separate fracture; n = 17)				
Floor	112.7 (69.7)	144.9 (111.1)	27.0	0.44
Medial wall	30.8 (44.8)	40.5 (27.9)	32.0	0.77
Floor continuous with medial wall (n = 2)	373.9 (NA)	NA	NA	NA
**Muscle change ratio**				
ID 10	0.5 (0.2)	0.5 (0.3)	329.0	0.34
ID 16	0.8 (0.5)	0.8 (0.5)	346.5	0.27
ID 11	0.6 (0.3)	0.7 (0.5)	284.5	0.91
ID 9 & 15	0.5 (0.1)	0.5 (0.3)	89.0	0.96

Note: IQR = interquartile range; NA = not available.

#### Inferior and medial rectus muscle changes and PE

Muscle changes were observed in 16.1% of subjects presented with a PE. Pearson Chi square did not show statistically significant difference between muscle change and PE in (Pearson Chi Square, *p* > 0.05) (Table 2) and between muscle change ratio and PE (Pearson Chi Square, *p* > 0.05) (Table 3).

### Multivariate analysis

#### Predictors of PE

Five independent variables were selected for this analysis: muscle change, number of fracture (single or double), internal orbital structures, fracture size (≤150 mm^2^ or >150 mm^2^) and fracture location. In addition, patients’ demographics, i.e. age (35 years old or more and less than 35 years old), gender and ethnic group (Malay and non-Malay) were added as the potential confounders.

After controlling for age, patients with orbital fracture size of more than 150 mm^2^ were three times more likely to exhibit a PE (adjusted odds ratio (AOR) = 3.01 (95% CI 1.17–7.92) (*p* < 0.05) (Table 4).

**Table 4:** Adjusted association between fracture size and PE.

Post-traumatic enophthalmos	
Characteristics	OR (95% CI)
**Fracture size**	
≤ 150 mm^2^	1.00
>150 mm^2^	3.01 (1.17–7.92)

Note: Hosmer-Lemeshow (Χ^2^(2) = 0.025, *p* = 0.99); % correctly classified = 70.1%, sensitivity = 31.0%, specificity = 89.7%.

## Discussion

MVA commonly involves young patients in this country [28] and this finding was consistent with other studies [29–35]. Having a PE particularly at a young age could lead to declining in quality of life due to the adverse psychological and cosmetic issues.

To date, the role of early CT scan to diagnose a PE has not been reported. Previous studies on muscle change [19,20,36], fracture size [18,37–38] and site [9,39–41] used delayed CT scans. The use of an acute CT to determine the PE may be misleading as swelling of the soft tissue, stranding of the fat layers and intramuscular hematoma may render radiographic interpretation [42]. Due to this equivocal characteristics, we used the clinical PE as the dependent variable in this study. However, we believe that early CT does offer some form of benefit in the diagnostic phase of this fracture. There are several characteristics on the CT scan which is clinically stable in both the acute and delayed conditions and may be used as a reliable predictor. These characteristics involve the bony structures. This finding is important as we may now able to predict a PE using the acute CT scan.

Acute CT scan imaging is required for pre-operative assessment [43–47] and surgical intervention is recommended early in some conditions such as the oculocardiac reflex, foreign body and others [42]. If a PE could be corrected simultaneously during the early repair, this would benefit the patient from having a secondary repair at a later date. Early surgery is proposed as it results in better recovery and good prognosis [48].

We found that the combination fracture of the posterior ledge and inferior orbital fissure is substantial. However, when evaluated individually, the structure was less likely to be involved due to their small bony features compared to the adjacent orbital floor and even the lateral wall [24] Thus, the non-significant association of the individual internal orbital structure with PE is justifiable.

Non-statistically significant associations were also observed between the fracture location, size and muscle change with PE. Nolasco and Mathog (1995) found that PE was significantly more prominent in patients with combined medial-inferior orbital wall fractures compared to those with pure medial wall fractures [41]. Similar observation was reported by Burm et al. (1999) [9]. In addition, He et al. also reported more cases of PE were observed significantly in patients with both orbital medial wall and floor fractures compared to those with floor only fractures [40]. Sung et al. (2013) observed that PE of more than 2 mm were apparent with a fracture size of at least 190 mm^2^ or larger [38].

Following a multivariate analysis, we showed that the single most important factor to predict a PE in the acute phase was the fracture size. A PE is three times more likely to be observed in pure orbital blowout fracture in patients presented with fracture size of more than 150 mm^2^ compared to those with less than 150 mm^2^ in size. This fracture size documented in this study was smaller the size reported by Sung et al (2013) [38]. The fracture size will meaningfully address the PE assessment whilst the combination of the internal orbital structures add some new information to the current literature on this subject matter. This will allow the surgeons to plan their intervention strategies accordingly as early surgical repair is paramount for better surgical outcome [37, 49].

The role of the fracture size as the predictor needs to be validated by comparing it in the acute and delayed CT scans. Likewise with the other potential factors especially the soft tissue characteristics during the acute and delayed phases.

### Limitations

Among the limitations encountered in this study was the absence of subsequent CT scans particularly in delayed setting. This is a common practice in this country where a CT is only performed at the initial stage of hospitalization. In addition, due to the same factor, our inclusion criteria described the need of a CT of 2 mm slice and less. An ideal imaging would be a high resolution CT or HRCT of the orbit with 1 mm slice, but unfortunately, many patients were subjected to only one CT (many had a simultaneous CT brain and orbit) and the slice was 2 mm.

In addition, incomplete documentation of the injury and related information was not uncommon.

## Conclusion

Fracture size larger than 150 mm^2^ was a radiological predictor of PE. Additionally, concurrent posterior ledge and inferior orbital fissure fractures may also contribute to PE.

## Supporting information

S1 Table Study dataset. (SAV)
